# Principal component analysis to identify the major contributors to task-activated neurovascular responses

**DOI:** 10.1016/j.cccb.2022.100039

**Published:** 2022-01-15

**Authors:** James Ball, Ronney B Panerai, Claire A.L. Williams, Lucy Beishon

**Affiliations:** a University of Leicester, Department of Cardiovascular Sciences, Leicester, UK; b NIHR Leicester Biomedical Research Centre, British Heart Foundation Cardiovascular Research Centre, Glenfield Hospital, Leicester, UK

**Keywords:** Neurovascular coupling, Dementia, Alzheimer's disease, Cerebrovascular response

## Abstract

**Background:**

Consensus on the optimal metrics for neurovascular coupling (NVC) is lacking. The aim of this study was to use principal component analysis (PCA) to determine the most significant contributors to NVC responses in healthy adults (HC), Alzheimer's disease (AD), and mild cognitive impairment (MCI).

**New method:**

PCA was applied to three datasets: 1) 69 HC, 2) 30 older HC, 34 AD, and 22 MCI, 3) 1&2 combined. Data were extracted on peak percentage change in cerebral blood flow velocity (CBFv), variance ratio (VR), cross-correlation function peak (CCF), and blood pressure, for five cognitive tasks. An equamax rotation was applied and factors were significant where the eignevalue was ≥1. Rotated factor loadings ≥0.4 determined significant NVC variables.

**Results:**

PCA identified 12 significant factors accounting for 78% of variance (all datasets). Contributing variables loaded differently on the factors across the datasets. In datasets 1&2, peak percentage change in CBFv contributed to factors explaining the most variance (45–58%), whereas cognitive test scores, fluency and memory domains contributed the least (15–37%). In the combined dataset, CBFv, CCF and fluency domain contributed the majority (33–43%), whereas VR and attention the least (6–24%).

**Conclusions:**

Peak percentage change in CBFv and the visuospatial task consistently accounted for a large proportion of the variance, suggesting these are robust NVC markers for future studies.

## Highlights

• A range of metrics are available to measure to cerebrovascular responses to task activation.
• We used principal component analysis to reduce dimensionality in a large dataset and determine physiological variables with the greatest contribution to the cerebrovascular response.
• Peak percentage change in cerebrovascular response was a consistent marker across datasets and the visuospatial task contributed the most variance.
• There was limited overlap between cognitive tasks and domains suggesting lack of redundancy in the data.

## 1 Introduction

Neurovascular coupling (NVC) is the relationship between cerebral blood flow (CBF) and local neural activity in the neurons and glia (oligodendrocytes, astrocytes and microglia) in the neurovascular unit, which can be triggered by various stimuli [10]. In the majority of healthy individuals, an increase in neural activity leads to an increase in CBF, owing to a combination of the increased metabolic and energy demands and the relatively small energy storage capacity of the brain [2]. NVC is regulated via a selection of factors including metabolic, myogenic, autonomic and sheer wall stress regulation [18].

There are 50 million people living with dementia worldwide [16]. Despite an increasing proportion of the population living with dementia, there are surprisingly few diagnostics and treatments available. Dementia can cause uncoupling of the NVC processes described above, resulting in an impaired hyperaemic response when individuals are cognitively challenged [4, 23]. The observed reduction in CBF [8] and NVC results in a mismatch between CBF demand and supply, leading to cognitive dysfunction [14]. Proposed by Zlokovic et al, the two-hit vascular hypothesis suggests that the amyloid cascades typical in Alzheimer's disease (AD) are initially triggered by vascular events and risk factors (e.g. diabetes and hypertension) [25]. In the first hit of the model, factors such as atherosclerosis trigger vascular dysfunction in the form of a reduced vascular integrity [14].  This results in a reduction in CBF leading to neuronal dysfunction and eventually dementia symptomatology [14]. The second hit of the model, features a rise in amyloid-beta (Aβ), as a result of reduced clearance, and enhanced tau hyperphosphorylation [12,14].

AD is the most common form of dementia (∼60%), closely followed by vascular dementia (VaD) (∼20%) [20]. Risk factors such as atherosclerosis and cerebral small vessel disease trigger a decrease of vascular integrity and, in turn, a reduction in CBF, resulting in a mis-match between CBF and neural activity [9]. VaD secondary to cerebral small vessel disease is associated with chronic hypoperfusion and increased vascular resistance which inherently limits the brain's capacity to meet the metabolic demands of increased cognitive activity [9,11]. In a study by Asil and Unzer, only participants with VaD had reduced CBFv responses to visual stimulation compared to participants with AD [1]. Fewer studies have investigated rarer dementia sub-types (Parkinson's disease dementia, Lewy body dementia), although one study demonstrated no differences in NVC between healthy controls and people with Parkinson's disease with and without dementia [19].

Non-invasive methods are particularly attractive to study NVC in dementia owing to their acceptability and tolerability compared to alternatives. One such method uses transcranial Doppler ultrasonography (TCD) and typically measures the peak percentage change in CBF velocity (CBFv) at rest and following cognitive stimulation [22]. Other common parameters include: area under the curve of the CBFv response, time to peak response, and more recently dichotomisation into the presence (“responders”) and absence (“non-responders”) of a response [5]. Typically, repeated trials are used to improve the signal to noise ratio, but these are limited by protocol duration, and thus tolerability when translating to clinical practice. More recent protocols have demonstrated that single trials are feasible and produce robust NVC responses [6], however, “non-responders” to stimulation are often present even in healthy populations [5]. Whilst, response rate can discriminate between dementia and healthy ageing with good accuracy [3], this relies on the use of multiple tasks and physiological parameters, which may not be easily translated to a clinical setting.

There remains a lack of consensus on the most appropriate measurement technique to use in NVC studies in both healthy and disease states [21]. Given the multitude of available techniques, reducing dimensionality in the data may be useful to identify the most important metrics that account for the largest proportion of the explained variance. Principal component analysis (PCA) is one such method of reducing the dimensionality in large datasets. This can be achieved by eliminating redundant information in the dataset, and retaining orthogonal variables, thus reducing multi-collinearity.  Although a recent analysis demonstrated good discriminative ability of NVC between AD, mild cognitive impairment (MCI), and healthy older adults (HC) [3], this relied upon data from two metrics across five tasks, which may not always be practical to collect. Thus, PCA may be able to reduce the need for multiple tasks and trials by identifying the most useful variables for discrimination.

Therefore, the aim of this analysis was to identify the most useful NVC metrics from a large, healthy dataset of 45 variables using PCA, and to compare these with a second data including participants with a diagnosis of AD or MCI.

## 2 Methods

### 2.1 Participants

The data in this study were extracted from two datasets in the Cerebral Haemodynamics in Ageing and Stroke Medicine (CHiASM) database. The first dataset was a sample of 69 healthy volunteers (HC), free from major co-morbidities or medications that adversely affect cognitive function. The second dataset included 56 patients with a diagnosis of AD or mild cognitive impairment (MCI), and 30 matched healthy controls recruited from May 2017 – January 2020. The NIA/AA 2011 criteria were used to confirm a diagnosis of AD or MCI in the original studies. Inclusion, exclusion criteria and research protocols were similar across the original studies and can be seen in our previous publications [3, 4, 6]. In brief, exclusion criteria were as follows: under the age of 18, pregnant, planning pregnancy or lactating, severe respiratory disease, severe cardiac failure with an ejection fraction of <20%, severe carotid artery stenosis and generally unable to comply with the requirements of the study. Participants on anti-dementia medications were suitable for inclusion in all studies. HC participants were without major comorbidity, measurable cognitive deficit or medication known to have an adverse effect on cognitive function. Well-controlled or stable comorbidities (e.g. hypertension, diabetes) were suitable for inclusion. Written, informed or personal consultee consent was sought from all participants used in the original studies, and all studies had University of Leicester, or Research Ethics Committee approval (references: 17/WA/0089 and 18/YH/0396). All studies were conducted in accordance with the Declaration of Helsinki 1964.

### 2.2 Data collection protocol

The data used in this study were collected at the CHiASM research space, which is a quiet, temperature-controlled environment. Participants were requested to abstain from stimulants (caffeine), depressants (alcohol), nicotine, large or heavy meals and strenuous exercise for at least 4 hours previous to the measurements being taken. Continuous TCD (DWL Doppler Box or Viasys Companion III), measurements were taken for CBFv via bilateral insonation of the middle cerebral arteries (MCAs). In addition, continuous measurements of heart rate (HR, using a 3-lead ECG), beat-to-beat blood pressure (BP, using a Finometer by Finapres Medical Systems) and end-tidal CO_2_ (ETCO_2_, using capnography - Capnocheck Plus) were recorded alongside CBFv. The signals were sampled at 500 samples/s before being stored in a data acquisition system (PHYSIDAS, Department of Medical Physics, University Hospitals of Leicester NHS Trust), for offline analysis.

Each participant was asked to complete five minutes of rest (baseline), followed by five cognitive tasks (attention, fluency, language, visuospatial, and memory) from the Addenbrooke's Cognitive Examination-III (ACE-III). Prior to, and between each cognitive task, there was a 1-minute rest period in order for CBFv to return to baseline. The start of each task was marked with an event recorder. We extracted the following parameters from the database for the final datasets: CBFv, BP, HR, and ETCO_2_ during cognitive stimulation. We also extracted data on key demographics (e.g. age and sex), and cognitive test scores.

Once collected, the data was processed and non-physiological spikes were removed by linear interpolation. The data were then passed through a median filter to remove smaller spikes and a zero-phase Butterworth filter was used to low-pass filter the data at 20 Hz. Handedness (Edinburgh Handedness Inventory [15]) was assessed to classify participants as right or left hand dominant, and thus the corresponding hemisphere as dominant and non-dominant.

Peak percentage change from a twenty second baseline period prior to task activation in CBFv, and BP was extracted. Peak percentage change was calculated at two time points: T2 (5 seconds after activation as the initial response), and T3 (10 seconds after activation as the sustained response). This allowed uniform measurements for percentage change.

In addition to data on peak percentage change, we extracted data on the cross-correlation function peak (CCF), and the variance ratio (VR). CCF is the time dependant cross-correlation between a population coherent average and an individual participant's signal, with the highest CCF possible being 1. The VR compares the variability of the signals before and after the NVC stimulus, where a higher VR represents greater (expected) variability in the signal post-activation.

### 2.3 Data Analysis Methods

#### 2.3.1 Principal component analysis

PCA was performed to identify the NVC metrics which provide the most relevant information or loadings. We tested PCA in the combined dataset, healthy dataset and the patient dataset, to identify if different variables contributed to a greater proportion of the variance in different populations. The following variables were investigated: peak percentage change in CBFv for both hemispheres at T2 and T3, peak percentage change in MAP, CCF and VR (all for five cognitive tasks listed above), and the sub-domain test scores of the ACE-III (attention, language, verbal fluency, visuospatial, memory). We used an equamax rotation assuming orthogonal variables and factors which we confirmed by the correlation matrix. We determined significant factors to be those with an eigenvalue of greater than 1, and visually inspected this on a scree plot. We determined significant variables to be those which loaded the highest on each factor with an eigenvalue above 1. All analyses were performed in SPSS version 26 For Windows. Supplemental data can be accessed here: https://figshare.com/s/b8285455f379761f9e8a.

## 3 Results

### 3.1 Demographics

Table 1 shows the demographics of the two data-sets (full list published in [3]). Education level was lower amongst AD participants, with higher anti-dementia drug use, lower resting CBFv, BP, and cognitive test scores. HC were younger than those with MCI or dementia in the patient data-set (67 vs 72 years old, respectively).

**Table 1:** Demographics of the two datasets (healthy and patient - participants with Alzheimer's dementia [AD] or mild cognitive impairment [MCI]). Data are mean (standard deviation), median [inter-quartile range], or number (percentage). Significance testing by chi-square (nominal data), one-way ANOVA (normally distributed, continuous), or Kruskall-Wallis (non-normally distributed, continuous). Significant where p<0.05, highlighted in bold. Abbreviations: ACHeI: acetylcholinesterase inhibitor, CBFv: cerebral blood flow velocity, ETCO_2_: end-tidal CO_2_, BP: blood pressure, ACE-III: Addenbrooke's Cognitive Examination III.

Demographic	Healthy control	MCI	AD	P value
Healthy data-set				
n	69	-	-	
Mean age (years)	49 (20)	-	-	
Female Sex (n, %)	40 (58)	-	-	
Right handed (n, %)	63 (91)	-	-	
Patient data-set				
n	30	22	35	
Mean age (years)	67 (8.6)	72.3 (8.5)	72.2 (8.9)	0.033
Female (n, %)	13 (43.3)	4 (18.2)	12 (34.3)	0.16
Education (years)	18 [16–20]	15 [12–17]	14 [11.25–17]	0.009
ACHeI (n, %)	0 (0)	5 (22.7)	23 (65.7)	<0.005
CBFv non-dominant (cm/s)	50.7 (9.3)	46.7 (7.2)	44.0 (9.4)	0.012
CBFv dominant (cm/s)	51.6 (7.8)	46.4 (7.2)	42.9 (11.3)	0.001
ABP (mmHg)	98.6 (12.8)	92.4 (11.1)	95.2 (22.4)	0.001
Heart rate (bpm)	68.1 (13.5)	62.9 (11.5)	63.1 (11.1)	0.18
ETCO_2_ (mmHg)	37.6 (3.1	36.1 (3.9)	35.0 (3.2)	0.01
ACE-III total score	98 [95–99]	87 [85–92.8]	77 [70–82]	<0.005
Attention	18 [17.3–18]	17 [16–18]	15 [12–16.5]	<0.005
Memory	25.5 [24.3–26]	22 [19–24]	15 [11.5–17.5]	<0.005
Fluency	13 [11.3–14]	11.5 [9–12]	9 [7–10]	<0.005
Language	26 [26–26]	25 [25–26]	25 [24–26]	<0.005
Visuospatial	16 [15.3–16]	16 [15–16]	15 [13–15.5]	<0.005

### 3.2 PCA results

#### 3.2.1 PCA in healthy dataset

**Fig. 1:**
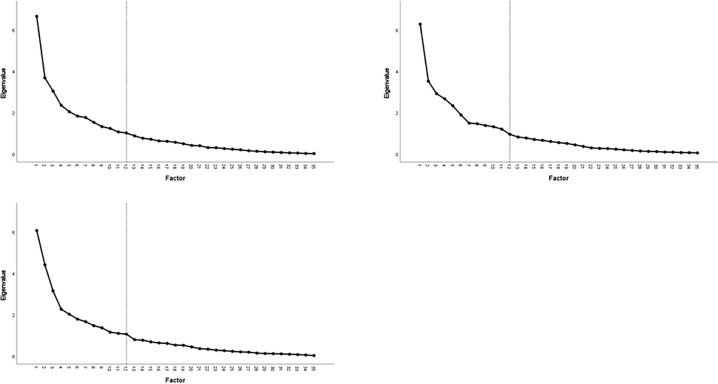
PCA.

In preliminary analyses, data from both hemispheres loaded on the same factors, providing no additional information, and so were averaged across hemispheres. This left 35 variables (averaged CBFv and BP at T2 and T3, VR, CCF, for five cognitive tasks and their corresponding test scores) for analysis. In the healthy dataset, 12 components were identified with an eigenvalue ≥1 which accounted for 78.6% of the total variance (Table 2, Fig. 1a). The first factor accounted for almost a fifth (19%) of all the variance (Table 2). Supplementary Table 3 shows the variables which load on the rotated components, accounting for the majority of the variance in the data. The first factor was predominantly loaded by visuospatial variables (CBFv, CCF).

**Table 2:** The total variance explained for the averaged healthy dataset, with corresponding eigenvalues and the cumulative percentage of the variance for all twelve factors.

Factor	Eigenvalue	% of variance	Cumulative %
1	6.6	19.0	19.0
2	3.7	10.5	29.5
3	3.0	8.7	38.2
4	2.4	6.7	44.9
5	2.0	5.8	50.7
6	1.8	5.2	55.9
7	1.8	5.0	61.0
8	1.5	4.4	65.3
9	1.3	3.8	69.1
10	1.2	3.5	72.6
11	1.1	3.1	75.7
12	1.0	2.9	78.6

In terms of the pattern of loadings, variables tended to load by task (2: language, 3: memory, 6: fluency, 8: attention). BP from different task loaded on the same factors (4 and 5), and VR loaded predominantly on factor 7. This suggests that tasks provide different information, but within tasks individual variables may not provide additional information. Similarly, BP and VR do not provide additional information amongst the various tasks.

#### 3.2.2 PCA in patient data-set

In the patient dataset, 12 factors were identified with an eigenvalue at or close to 1, accounting for 78.2% of the total variance (Table 3, Fig. 1b). In keeping with the healthy results, the first factor accounted for the majority of the variance (17.9%). Supplementary Table 2 shows the variables which load on the rotated components. In contrast to the healthy dataset, the majority of variables (n=18) loaded on the first factor, and were predominantly CBFv, BP and CCF variables across the five tasks. Test scores mainly loaded on factors 2 and 3. In contrast to the healthy dataset, VR from different tasks did not load together on one factor, and variables did not load within task onto individual factors (Supplementary Table 2).

**Table 3:** The total variance explained for the averaged patient dataset, with corresponding eigenvalues and the cumulative percentage of the variance for all twelve factors.

Factor	Eigenvalue	% of variance	Cumulative %
1	6.3	17.9	17.9
2	3.5	10.0	27.9
3	2.9	8.3	36.3
4	2.7	7.6	43.9
5	2.3	6.7	50.5
6	1.9	5.4	55.9
7	1.5	4.3	60.2
8	1.5	4.2	64.3
9	1.4	3.9	68.3
10	1.3	3.8	72.0
11	1.2	3.4	75.5
12	0.95	2.7	78.2

#### 3.2.3 Combined dataset

In keeping with the patient and healthy datasets, the combined dataset had 12 significant factors (eigenvalue ≥1), and the first factor accounted for the majority of the variance (17.3%) (Table 4, Fig. 1c). However, when assessing the rotated factors and variable loadings the pattern was distinct from that seen in the previous two datasets. Factors 1 and 9 were loaded predominantly by memory test scores, and CBFv and CCF values were split across factors within their respective tasks (3, 5, 6, 8, and 12) (Supplementary Table 3). BP variables by task loaded predominantly on factors 2, 7 and 11, and the remaining VR variables loaded on factor 10. These results suggest independence of the CBFv data across different cognitive domains, when healthy and patient data are combined into one dataset.

**Table 4:** The total variance explained for the averaged patient dataset, with corresponding eigenvalues and the cumulative percentage of the variance for all twelve factors. .

Factor	Eigenvalue	% of variance	Cumulative %
1	6.3	17.9	17.9
2	3.5	10.0	27.9
3	2.9	8.3	36.3
4	2.7	7.6	43.9
5	2.3	6.7	50.5
6	1.9	5.4	55.9
7	1.5	4.3	60.2
8	1.5	4.2	64.3
9	1.4	3.9	68.3
10	1.3	3.8	72.0
11	1.2	3.4	75.5
12	0.95	2.7	78.2

#### 3.2.4 Comparison of contributing variables

Table 5 shows the factors against which variables and cognitive domains loaded and the variance explained by these factors. In the healthy and patient datasets, CBFv and the visuospatial domain loaded against factors explaining the most variance (45–58%), whereas cognitive test scores and the fluency and memory domains contributed the least (15–37%). In the combined dataset, CBFv, CCF and the fluency domain contributed the majority (33–43%), whereas VR and attention the least (6–24%).

**Table 5:** The factors and variance explained by variable and cognitive domain for each of the three datasets. CBFv= cerebral blood flow velocity, CCF= cross-correlation function peak, BP= blood pressure, VR= variance ratio.

Variables contributing most variance (high to low)	Factors loading	% variance explained by these factors	Cognitive domains contributing most variance (high to low)	Factors loading	%variance explained
Healthy					
CBFv	1, 2, 3, 4, 6, 8, 10	58	Visuospatial	1, 3, 5, 7, 8, 12	45.8
CCF	1, 2, 3, 6, 8	47.8	Memory	3, 5, 7, 9 10, 11	29.9
BP	3, 4, 5, 8, 9	29.4	Attention	4, 5, 7, 8, 12	24.8
VR	3, 7, 8, 11	21.2	Language	2, 4, 9, 11	24.1
Cognitive test score	5, 9, 11 ,12	15.6	Fluency	4, 5, 6, 7	22.7
Patients					
CBFv	1, 2, 3, 4, 5, 6	55.9	Visuospatial	1, 2, 3, 4 10, 11	51
BP	1, 2, 5, 6, 7, 9, 11	51.6	Attention	1, 2, 3, 5, 11	46.3
CCF	1, 4, 5	32.2	Fluency	1, 2, 3, 6, 7	45.9
VR	3, 7, 10, 11	19.8	Language	1, 2, 3, 7	40.5
Cognitive test score	2, 3	18.3	Memory	1, 3, 5, 9	36.8
Combined					
CBFv	3, 4, 5, 6, 8, 12	33.6	Fluency		1, 2, 6, 7, 10
CCF	3, 4, 5, 6, 8, 12	33.6	Visuospatial	1, 2, 4, 10	39.7
BP	2, 6, 7, 8, 11	29.7	Language	1, 5, 7	27.8
Cognitive test score	1, 9	21.2	Memory	1, 3, 9, 11	33.3
VR	10, 12	6.3	Attention	2, 8, 9, 12	23.7

## 4 Discussion

### 4.1 Summary of results

From a total of 35 potential markers of NVC efficacy, PCA revealed twelve significant factors for each of the three datasets (healthy, patient, and combined), which accounted for approximately 78% of the variance. Similarly, the first factor accounted for the majority of the variance in each of the three datasets (17–19%). However, the variables contributing to each factor differed considerably. In the healthy and combined datasets, variables within the same task (e.g. CBFv, CCF) tended to load on the same factor, suggesting minimal overlap in the variance explained between cognitive domains. However, this pattern was not replicated in the patient dataset suggesting greater overlap in the information provided by different cognitive domains.

### 4.2 Variable contributors to NVC responses

To our knowledge, this is the first study to use PCA to identify the relative contributions of different physiological variables derived from NVC metrics measured using cognitive paradigms in the MCA, from a large dataset of both healthy and cognitively impaired data. Previous studies have characterised NVC responses according to the percentage change, [6,22], presence and absence of response [3,5], time to peak [24], and subcomponents [13,17], but few have specifically examined the proportion of the variance explained by different physiological variables and the extent of redundancy is these variables. In a study by Chiarelli of NVC in mild AD as measured by EEG and fNIRS, PCA was used to provide a global EEG signal from timecourses which explained the majority of the variance for each frequency band [7]. However, the study did not specifically investigate or report which timecourses or physiological signals were redundant and could be eliminated for future studies, and the PCA approach was not extended to the haemodynamic data or responses. However, Squair et al studied data from 130 participants who underwent visual light stimulation with simultaneous recordings of CBFv in the posterior cerebral artery, and PCA was used as part of a larger network analysis to determine the most stable NVC metrics derived from the gain, rate time, and natural frequency of the NVC response [21]. Consistent with the findings recorded here, peak percentage change was found to be a highly stable marker of NVC, and accounted for the majority of explained variance in the dataset reported here. The findings reported in this study extend those by Squair et al by examining CBFv and physiological data obtained in the middle rather than posterior cerebral artery and using cognitive rather than visual stimuli across a range of five key domains. Furthermore, the data presented here include both healthy and cognitively impaired participants and we were able to identify differences in the pattern of variance explained by NVC metrics according to diagnosis. In the study by Squair et al, new NVC metrics derived from PCA and cluster analysis were able to discriminate between able bodied individuals and those with spinal cord injury, which were not identified using traditional NVC metrics. However, it remains unclear whether these variables which have been derived from the posterior cerebral artery using light stimulation can be applied to measurements taken in other vessels (e.g. anterior and middle), and other stimuli (e.g. cognitive, motor, sensory). The results from this analysis suggest that those based on the peak percentage change in CBFv are likely to be applicable to other datasets, but this remains to be formally tested. Interestingly, cognitive test scores contributed the least variance in two of the datasets, suggesting physiological parameters such as the peak percentage change may provide additional information on cerebral function beyond those routinely tested in the clinic. Of the cognitive domains studied, visuospatial was a consistent contributor across the three datasets, which is likely to reflect a robust and stable response across participants. However, in the combined and healthy datasets variables loaded onto factors separated by task, suggesting limited overlap between cognitive domains and tasks studied, indicating a lack of redundancy in data across cognitive domains. This may reflect that NVC responses occurring in different brain regions and cognitive domains have different physiological profiles, thus limiting their overlap in terms of variance in the dataset. CBFv and CCF tended to load on the same factors within tasks, and thus limited information is gained from combining CCF and CBFv metrics. However, VR and BP load on different factors from CBFv and CCF, providing potentially useful information beyond these variables. Finally, we included peak percentage change in CBFv and BP at two time points (T2 and T3), however these variables frequently loaded on the same factor and therefore using two time points is unlikely to provide benefit above using a single time frame of peak percentage change.

### 4.3 Limitations and future directions

This study was limited to a set of 35 NVC metrics and did not account for all available or previously studied markers of NVC (e.g. time to peak). Future studies could consider examining additional metrics beyond those included in this analysis. This study focussed on NVC responses obtained from the MCA using cognitive paradigms and it remains unclear how easily findings can be extrapolated to measurements taken in other vessels, using different types of stimuli. Finally, future studies should consider using the findings from this study to refine NVC metrics used in cognitive experiments, particularly to ensure protocols are tolerable for patients with cognitive dysfunction, simultaneously maximising information obtained whilst minimising protocol duration. The loadings from this study could be applied to future datasets to investigate the discriminant ability of the derived loadings between dementia and healthy ageing.

### 4.4 Conclusions

Using PCA, we identified which physiological variables from a larger physiological dataset that contribute the majority of variation in NVC data, and therefore may provide better information and reduce the volume of data collection in future protocols for clinical applications.

## Appendix Supplementary materials

Image, application 1
