# Supplementary material for: Principal component analysis to identify the major contributors to task-activated neurovascular responses
Source: Cereb Circ Cogn Behav. 2022 Jan 15;3:100039. doi: 10.1016/j.cccb.2022.100039 (PMC9616234; doi:10.1016/j.cccb.2022.100039)
Supplement: Supplementary file 1 [file mmc1.docx]

**Title**

Principal component analysis to identify the major contributors to task-activated neurovascular responses

**Authors**

James Ball^a^, Ronney B Panerai^a,b^, Claire A.L Williams^a^, Lucy Beishon^a^

**Affiliations**

a University of Leicester, Department of Cardiovascular Sciences, Leicester, UK

b NIHR Leicester Biomedical Research Centre, British Heart Foundation Cardiovascular Research Centre, Glenfield Hospital, Leicester, UK

**Corresponding author**

Dr Lucy Beishon

Room 419

Level 4, Robert Kilpatrick Clinical Sciences Building

Leicester Royal Infirmary

Leicester

LE1 5WW

Tel: 0116 252 3134

Email: lb330@le.ac.uk

| **Factor** | **Variables** | **Rotated loading** |
| --- | --- | --- |
| 1 | Visuospatial CCF  Visuospatial CBFv T2  Visuospatial CBFv T3 | 0.93  0.86  0.84 |
| 2 | Language CBFv T3  Language CCF  Language CBFv T2 | 0.86  0.78  0.75 |
| 3 | Memory CBFv T2  Memory CCF  Memory BP T2  Visuospatial BP T3  Memory VR | 0.80  0.80  0.63  0.42  0.40 |
| 4 | Language CBFv T2  Language BP T2  Language BP T3  Fluency BP T2  Attention CBFv T2  Attention BP T2 | 0.46  0.84  0.66  0.52  0.51  0.50 |
| 5 | Fluency BP T2  Attention BP T2  Fluency BP T3  Visuospatial BP T3  Visuospatial BP T2  Memory domain score | 0.46  0.46  0.72  0.69  0.69  -0.56 |
| 6 | Fluency CCF  Fluency CBFv T3  Fluency CBFv T2 | 0.87  0.75  0.69 |
| 7 | Fluency VR  Memory VR  Visuospatial VR  Attention VR | 0.72  0.66  0.61  0.60 |
| 8 | Visuospatial VR  Attention CBFv T3  Attention BP T3  Attention CCF | 0.45  0.83  0.67  0.48 |
| 9 | Memory BP T2  Memory BP T3  Language domain score | -0.45  -0.82  0.77 |
| 10 | Memory CBFv T3 | -0.87 |
| 11 | Memory domain score  Language VR | -0.54  0.87 |
| 12 | Visuospatial domain score  Attention domain score | 0.79  0.55 |

Supplementary Table 1. Rotated factors with variables loadings against each component accounting for the majority of the variance in the data (healthy dataset). Factors with loadings <±0.40 were considered non-significant and supressed for visualisation. CBFv= cerebral blood flow velocity, CCF= cross-correlation function peak, BP= blood pressure, VR= variance ratio.

| **Factor** | **Variables** | **Rotated loading** |
| --- | --- | --- |
| 1 | Language CBFv T2  Attention CBFv T2  Fluency CCF  Language CCF  Attention CCF  Fluency CBFv T2  Language BP T2  Language CBFv T3  Attention CBFv T3  Language BP T3  Attention BP T3  Visuospatial BP T3  Attention BP T2  Fluency BP T2  Visuospatial BP T2  Memory BP T2  Memory CBFv T3  Fluency CBFv T3 | 0.74  0.70  0.65  0.61  0.60  0.58  0.55  0.55  0.51  0.50  0.47  0.44  0.43  0.50  0.45  0.42  0.41  0.47 |
| 2 | Language CBFv T3  Attention BP T2  Fluency BP T2  Visuospatial BP T2  Fluency test score  Language test score  Attention test score  Visuospatial test score  Fluency BP T3 | 0.43  -0.60  -0.51  -0.46  0.48  0.41  0.53  0.41  -0.48 |
| 3 | Fluency test score  Language test score  Memory CBFv T3  Attention test score  Visuospatial test score  Memory VR | 0.70  0.68  -0.60  0.55  0.49  -0.42 |
| 4 | Visuospatial CBFv T2  Visuospatial CBFv T3  Visuospatial CCF | 0.77  0.77  0.73 |
| 5 | Attention CBFv T3  Memory ABP T2  Memory CBFv T2  Memory CCF  Memory ABP T3 | -0.41  0.53  0.50  0.44  0.50 |
| 6 | Fluency CBFv T3  Fluency ABP T3 | 0.58  0.53 |
| 7 | Language ABP T3  Fluency VR | 0.50  -0.55 |
| 8 | - | - |
| 9 | Memory ABP T3 | 0.68 |
| 10 | Visuospatial VR | 0.54 |
| 11 | Visuospatial ABP T3  Attention VR | -0.44  0.55 |
| 12 | - | - |

Supplementary Table 2. Rotated factors with variables loadings against each component accounting for the majority of the variance in the data (patient dataset). Factors with loadings <±0.40 were considered non-significant and supressed for visualisation. CBFv= cerebral blood flow velocity, CCF= cross-correlation function peak, BP= blood pressure, VR= variance ratio.

| **Factor** | **Variables** | **Rotated loading** |
| --- | --- | --- |
| 1 | Fluency test score  Visuospatial test score  Memory test score  Language test score | 0.91  0.87  -0.71  -0.64 |
| 2 | Visuospatial BP T2  Visuospatial BP T3  Attention BP T2  Attention BP T3  Fluency BP T2 | 0.76  0.75  0.72  0.56  0.43 |
| 3 | Memory CBFv T2  Memory CCF  Memory VR  Memory CBFv T3 | 0.90  0.86  0.60  0.56 |
| 4 | Visuospatial CCF  Visuospatial CBFv T3  Visuospatial CBFv T2 | 0.88  0.86  0.86 |
| 5 | Language CCF  Language CBFv T3  Language CBFv T2 | 0.84  0.83  0.72 |
| 6 | Fluency CBFv T2  Fluency CBFv T3  Fluency CCF  Fluency BP T3  Fluency BP T2 | 0.78  0.78  0.77  0.46  0.44 |
| 7 | Language BP T2  Language BP T3  Fluency BP T2 | 0.82  0.76  0.47 |
| 8 | Attention BP T3  Attention CBFv T3  Attention CCF  Attention CBFv T2 | 0.54  0.87  0.69  0.48 |
| 9 | Memory test score  Attention test score  Language test score | 0.63  0.91  0.70 |
| 10 | Visuospatial VR  Fluency VR | 0.83  0.76 |
| 11 | Memory BP T3  Memory BP T2 | 0.89  0.65 |
| 12 | Attention VR  Attention CCF  Attention CBFv | 0.45  0.44  0.82 |

Supplementary Table 3. Rotated factors with variables loadings against each component accounting for the majority of the variance in the data (combined dataset). Factors with loadings <±0.40 were considered non-significant and supressed for visualisation. CBFv= cerebral blood flow velocity, CCF= cross-correlation function peak, BP= blood pressure, VR= variance ratio.
